# Classical Pitfalls in Contemporary Nuclear Data Analysis

**DOI:** 10.6028/jres.093.124

**Published:** 1988-06-01

**Authors:** K. Heydorn

**Affiliations:** Isotope Division, Risø National Laboratory, DK-4000 Roskilde, Denmark

Nuclear methods of analysis are important as an alternative to chemical methods, and often serve as reference methods, because they rely on entirely different principles, associated with the atomic nucleus rather than the electronic configuration of the elements. Although a variety of nuclear analytical methods are found, they almost invariably resort to the process of counting for the final measurement. Sophisticated electronic equipment for counting, as well as associated program systems for data processing, are now commercially available as integrated units, and most analytical chemists will have to use these systems as “black boxes.”

Under proper conditions these systems are a great boon to practitioners of nuclear analytical methods, because they extract the maximum possible information from the data. However, it is important to be aware of their limitations, which may give rise to erroneous results without warning.

A recent example is the determination of Zn in a BCR candidate reference material RM 279 Sea Weed by a variety of methods, including instrumental neutron activation analysis. Only highly experienced laboratories from the European Community were invited to participate in the certification, and even then the results by INAA ranged from 48 to 57 mg/kg without any overlap between laboratories.

Such discrepancy is unacceptable for a well established analytical technique such as INAA with a reputation for being without significant systematic errors. Unfortunately this example is not unique, and an attempt is therefore made in what follows to point out possible pitfalls or sources of error that might be overlooked in contemporary nuclear analytical methods.

## Calibration Errors

Direct comparison between a sample and the corresponding primary standard, subjected to exactly the same treatment, is necessary to achieve the highest accuracy in the determination of an element [1]. In radiochemical neutron activation analysis it is possible to transform the sample so that it becomes identical with the comparator with respect to physical form, shape, self-absorption and other factors affecting the calibration: in INAA this is obviously not possible.

Instead it becomes necessary to correct the calibration for differences between sample and standard, and to select counting conditions where these corrections are as small as possible. Failure to do this may lead to considerable systematic error.

The use of substances of ill-defined or unknown composition as comparator standards may lead to gross errors; only pure elements or compounds with known stoichiometry should be used. Reference materials, whether certified or not, do not serve as calibrants, but only for the purpose of checking calibrations.

Calibration based on elements other than those to be determined is in principle possible, when the nuclear parameters are taken into account by the *k*_0_-factors [2]. This eliminates the need to have primary standards of all elements available in the laboratory, but failure to correct for differences between the actual conditions of measurement and those used in the determination of the *k*_0_-factors may lead to systematic errors.

One such example is the use of *k*_0_-factors for the determination of selenium using ^75^Se as indicator isotope, where failure to make coincidence corrections in close counting geometry leads to significant errors; such correction is of course taken into account automatically when a ^75^Se comparator standard is counted in the same counting position as the sample.

Even in an apparently simple case of chromium in zircalloy, where the ^51^Cr has only a single photopeak, and where the matrix element is used as a comparator, systematic errors are still difficult to eliminate in the *k*_0_-method.

## Identification Errors

Neglecting to identify all photopeaks in a spectrum may lead to serious systematic errors, because without such information it is impossible to ascertain the lack of interference needed to determine an element using a single photopeak.

Such interferences are particularly dangerous when determining elements with indicator isotopes having only one strong *γ*-line, e.g., Cr, Zn, and Hg. While interferences of ^75^Se on ^203^Hg and ^46^Sc on ^65^Zn are well-known, interference from ^152^Eu on ^65^Zn is often not considered and has in the past caused significant errors in some types of sample.

The reverse situation may also apply, when an automatic resolution of double peaks seems to indicate the presence of a close-by *γ*-ray in a perfectly pure spectrum. The number of counts in the photopeak proper is thus reduced, resulting in reported results being too low.

## Photopeak Area Evaluation Errors

*A priori* information on the presence or absence of interference in the processing of *γ*-spectra is a prerequisite for obtaining accurate results. However, even when an absolutely pure photopeak is at hand, peak areas are evaluated differently by different calculation methods particularly if the peak-to-base ratio is small.

The use of partial peak areas (PPA) to obtain optimum precision [3] also improves the accuracy when the same peak fraction is used for the comparator of the same element. In *k*_0_-calibration only total peak areas (TPA) are applicable, and the ratio TPA/PPA varies from 1.00 to 1.26 [4] depending upon the magnitude of the peak.

The use of least-squares fitting methods appears to solve this problem, but the calculated peak area still depends on the functional representation of the peak used in the program. This assumes importance in the evaluation of the reference pulser peak used to correct for dead-time and pile-up losses during counting. This peak is not Gaussian-shaped, and the channel contents are not governed by the Poisson statistics; an attempt to evaluate this peak by least-squares fitting of a model peak therefore leads to strong bias.

Resolution of doublets leads to different results, depending on whether the program is based on consultation with a library or not [5]. The first category has a greater risk of leading to errors of the second kind, as mentioned in the preceding paragraphs, whereas the second category is more likely to fail to discover the presence of small or closely lying interfering peaks. For closely located peaks that are not resolved, some programs do not calculate the combined peak areas, but may yield completely false results.

The only way of detecting these or other systematic errors in the analysis of nuclear spectrometry data is to apply at least two independent methods and only release results when these methods concur.
